# Editorial: Microglia and peripheral immune cells in aging and neurodegenerative diseases

**DOI:** 10.3389/fcell.2023.1178496

**Published:** 2023-03-14

**Authors:** Yiguo Qiu, Xianli Shen, Heping Xu

**Affiliations:** ^1^ Department of Cancer Immunology and Virology, Dana-Farber Cancer Institute, Boston, MA, United States; ^2^ Department of Immunology, Harvard Medical School, Boston, MA, United States; ^3^ Wellcome-Wolfson Institute for Experimental Medicine, School of Medicine, Dentistry and Biomedical Sciences, Queen’s University Belfast, Belfast, United Kingdom

**Keywords:** microglia, peripheral immune cells, brain, retina, aging, neurodegenerative disease, neuroinflammation

In contrast to the long-withheld concept that neurodegenerative diseases are “neuron-centric” disorders, emerging evidence from recent years has shifted this notion into a more “immune-centric “view highlighting the contribution of dysregulated immune responses to disease progression (Bido et al., 2021; Qiu et al., 2023). Microglia are principal resident immune cells of the central nervous system (CNS) whose dysregulation contributes to neurodegenerative diseases directly *via* promoting neuroinflammation or indirectly in response to cues from the peripheral immune system. Dysregulated immune responses correlate closely with CNS aging and neurodegenerative pathology. However, the heterogeneous nature of microglia and peripheral immune cells has impeded the characterization of their role and crosstalk in these processes. A comprehensive understanding of how peripheral immune cells act in concert with microglia to regulate the aging process and neurodegenerative diseases will advance the understanding of immunoregulation of neurological disorders and provide novel insights in the development of next-generation immunotherapies for various age-related neurodegenerative diseases.

In this Research Topic, we present a Research Topic of five articles describing different aspects of microglial and peripheral immune response in various age-related neurodegenerative diseases, including Alzheimer’s disease (AD), Parkinson’s disease (PD) and age-related macular degeneration (AMD). The two original research articles provide evidence of the contribution of microglial and peripheral proinflammatory responses to the neuropathology of AD and PD, respectively. Whereas the three review articles describe a general picture of brain and peripheral innate immune system in regulating neurodegenerative diseases affecting the brain and retina.

Neuroinflammation caused by sustained microglial inflammatory response is a hallmark shared by a series of neurodegenerative diseases and represents a key element that directly drives disease progression. In this Research Topic, Li et al. described that Acteoside (ACT), a neuroprotective compound, played a beneficial role in AD by suppressing microglial polarization towards a pro-inflammatory phenotype. Mechanistic analyses integrating multi-omics database have revealed that ACT regulates microglial polarization though oppositely modulating NF-κB and AMPK pathways. These findings suggest ACT as a potential therapeutic agent for neurodegenerative disease associated with neuroinflammation. In addition to neuroinflammation, peripheral proinflammatory response may be implicated in the pathogenesis of neurodegenerative diseases as well. Fu et al. reported an elevated level of IL-6 and TNF-α production in the sera of PD patients and the level correlates strongly with clinical symptoms. Noteworthy, activated microglia also produce IL-6 and TNF-α under pathologic conditions (Ishijima and Nakajima, 2021; Qiu et al., 2023). Perhaps the inflammatory factors detected in peripheral blood may reflect, in part, the leakage of such cytokines produced by inflamed microglia from degenerated brains. Therefore, the co-occurrence of dysregulated immune response of the periphery and brain may be a critical driving force of PD pathology.

As an essential component of the innate immune system, the NLRP3 inflammasome represents a molecular sensor of microglia that can be activated by stimuli of neurodegenerative diseases (Deora et al., 2020; Luciunaite et al., 2020). Autophagy, a self-degradative process that is pivotal in maintaining homeostasis, has been recently shown to play a vital role in regulating NLRP3 inflammasome in inflammatory disorders (Biasizzo and Kopitar-Jerala, 2020; Lu et al., 2022). Zhao et al. summarized recent advances on the role of autophagy in regulating NLRP3 inflammasome in a battery of neuroinflammatory diseases and discussed the underlying mechanism that provides theoretical insights for future research of inflammatory neurological diseases.

The immunoregulatory molecule phosphatidylinositol-specific phospholipase C gamma 2 (PLCγ2) is not only conventionally expressed by peripheral immune cells but also expressed by microglia exclusively in the CNS and plays an essential role in neuroinflammation (Koss et al., 2014; Jackson et al., 2021). Li et al. comprehensively reviewed the role of PLCγ2 and the relevant signaling pathways regulating AD progression and integrated the recent studies on the correlation of PLCG2 variants with this disease. Furthermore, the potentials and challenges of targeting PLCγ2 to treat AD were also discussed, which provides instructive guidance on the development of therapeutic strategies for AD.

As a part of CNS, the retina contains resident innate immune cells (microglia) and may also be populated with innate components from the periphery under pathological conditions. Dysregulated immune responses have been linked to retinal degenerative disorders, including AMD. A recent study provides an unparalleled depth of insight into key inflammatory pathways residing in microglia that promote neuroinflammation in AMD (Menon et al., 2019). Moreover, periphery immune response may also be essential in driven AMD. Brian et al. extensively reviewed the role of peripheral innate immune cells in AMD, highlighting the potential dysregulated crosstalk between immune cells residing in the retina and periphery. This review also discussed recent developments in single-cell transcriptomics that advance the understanding of developing therapeutic interventions for AMD targeting immune dysregulation.

In summary, the collection of articles in this Research Topic provides a snapshot of a newly rising multidisciplinary research field deciphering neurological diseases from the immunology perspective. These articles expand our understanding of central and peripheral immune responses in neurodegenerative diseases and shed light on developing therapeutic strategies targeting novel immunological targets. However, some questions which have gained increasing interest in this field remain to be further addressed. For example, as age is a main risk factor of many neurodegenerative diseases, is age-related dysregulation of microglial and peripheral immune response causally related to the onset or progression of neurodegenerative diseases? Whether and how microglia dialogue with peripheral immune cells and how such interaction affects disease progression? More importantly, the extreme heterogeneity of immune cells greatly impedes the accurate definition of their contribution to disease progression. Identification and characterization of distinct disease-associated immune cell subsets will surely favor the discovery of novel, effective therapeutic targets. We hope that the articles in this Research Topic will be insightful to a broad range of researchers working in the immune response to neurodegenerative diseases and will enlighten future research to decipher the role and molecular mechanisms at play between central and peripheral immune cells to regulate neurodegenerative diseases in both preclinical and clinical settings.
